# ESfix: An Embedded Program Repair Tool for Effective Removal of Concurrency Defects

**DOI:** 10.3390/e27030294

**Published:** 2025-03-12

**Authors:** Jingwen Zhao, Yanxia Wu, Yan Fu, Shuyong Liu

**Affiliations:** College of Computer Science and Technology, Harbin Engineering University, Nantong Street, Harbin 150001, China; zhaojw123@hrbeu.edu.cn (J.Z.); wuyanxia@hrbeu.edu.cn (Y.W.); liushuyong@hrbeu.edu.cn (S.L.)

**Keywords:** program repair, repair strategy, static analysis, semantic analysis, information entropy

## Abstract

Embedded programs are not only inseparable from our daily lives but are also widely used in aerospace, medical devices, and other fields that require very high security and stability. The uncertainty and randomness of the large amount of data generated by these systems during operation can be quantified by entropy. Traditional repair methods for concurrency defects may introduce new issues such as deadlocks, original semantic destruction, and high performance overhead. To overcome the limitations of the existing methods and help developers reduce the time and effort spent on fixing software defects, this paper proposes ESfix, a defect fixing technique applied to embedded software. ESfix first utilizes the bug information reported by the defect detection tool to locate the repair region and extracts the node information corresponding to the defective code. Then, ESfix optimizes the interrupt disable/enable strategies and lock strategies to repair data race and reduce bugs in information transmission, thereby reducing system entropy and improving data certainty and reliability. Finally, ESfix repairs atomicity violation defects using the reordering repair strategy, reducing information entropy by adjusting the order of information to ensure its integrity and consistency. ESfix conducts semantic analysis by analyzing the dependency graph in the control flow graph (CFG) to ensure that no new defects are introduced during the repair process, and to maintain the efficiency and accuracy of information transmission between different parts of the code. We evaluate the effectiveness of repair strategies through information entropy, and the experimental results show that ESfix not only improves performance but also reduces potential risks and losses.

## 1. Introduction

Concurrency bugs are difficult to detect, which further exacerbates the problem of writing correct concurrency source code since they may appear in only a small fraction of possible thread interleavings [1,2]. Defect detection techniques provide useful insights into the likely locations of concurrency defects, the inputs and states that caused bugs, and the abnormal operations that were performed during the bugs. However, successfully identifying concurrency bugs through tools or manually checking code does not necessarily mean that developers can immediately see the right repair. Being able to quickly self-repair defects after a defect failure has been detected is critical to providing comprehensive protection for embedded software.

Automated Program Repair (APR) aims to repair buggy programs by building source code level patches [3,4]. It is a painful, time-consuming, and expensive process, and a report by [5] shows that software debugging and repairing accounts for more than 50% of software development costs. For developers, manually repairing detected bugs during software development and maintenance is a very time-consuming and error-prone task [6,7]. To make matters worse, it is not uncommon to introduce violations of other critical attributes by eliminating concurrency defects as data race between memory accesses to the same location, such as potential deadlocks between threads. Therefore, the debugging process requires analyzing and understanding the failed execution, identifying the cause of the failure, implementing repairs, and verifying that the repaired program works correctly.

To repair concurrency defects, developers add, remove, and modify code fragments at specific locations to eliminate the root cause of concurrency bugs. Currently, most concurrency defect repair methods rely on generation-verification methods and semantics-driven methods [8,9]. Generation-verification-based repair methods [10,11,12,13] use fault location techniques to identify potential program locations that may cause bugs, generate a set of candidate patches, rank them, and modify the defective program. Finally, the modified program is compiled and verified by test cases. However, test cases cannot cover all paths and functions of the program, which can lead to a large number of overfitting patches. Semantics-driven-based methods [14,15,16,17,18] use program analysis techniques to obtain program execution information and path constraints, and formally encode the problem, enhancing these code regions by adding appropriate checks and synchronization mechanisms that may prevent concurrency problems. However, the method is prone to introducing another new bug.

Efficient and fully automated repair methods are not always available. Compared to other software, embedded software has higher requirements in terms of the application environment and the real-time, reliability, and design requirements, and usually uses an interrupt mechanism to realize the real-time concurrent response. Compared with multi-threaded programs, interrupts occur randomly and unpredictably, and their internal state is invisible to tasks and other interrupt handlers. Second, interrupts have asymmetric preemption relationships and different concurrency control mechanisms. Correctly repairing concurrency bugs in embedded software requires strong domain knowledge and technical background, as it involves shared resource locations, read/write operation locations, and the interleaving of interrupts and tasks, and often requires enforcing interrupt-specific synchronization operations [19,20]. Finding the correct location to insert an interrupt operation is a challenge when repairing an embedded program, as the correctness of the program semantics must be ensured [21]. The length of the synchronization critical region must also be considered, as long critical regions can lead to high complexity and resource overhead [22]. The existing methods for repairing thread-level concurrency bugs cannot be directly used to solve these problems.

In automatic program repair methods, mutual information can help identify which parts of the code are interdependent, which is crucial for understanding bug propagation and implementing repair strategies. We consider the program to be a source of information, where bugs and defects can be seen as noise in information transmission. One of the goals of program repair is to minimize this noise and improve the reliability and effectiveness of information transmission. To overcome the above two problems in embedded software, this paper proposes ESfix, our new methodology for concurrent defect repair built on embedded software bug reports that capture and efficiently locate the repair space. ESfix provides precise localization for most defective programs through static analysis, and utilizes dynamic and testing information from program execution to calculate the suspiciousness of different code segments. By calculating the entropy of different parts of the program, developers can identify the areas most likely to introduce bugs or defects, and make targeted repairs to avoid generating redundant code and making large-scale changes to the source code. Meanwhile, repair strategies are optimized to further improve the accuracy and efficiency of the repair, and the recommendations do not lead to serious performance degradation or code complexity. If the repair recommendations are too complex, ESfix will intentionally abort the bug repair and inform the developer of the relevant action.

Overall, this work has made the following contributions:

- We proposed new repair framework called ESfix, which aims to repair concurrency defects in embedded programs.
- We optimize the interrupt disable/enable strategy and the locking strategy to reduce the performance overhead.
- We optimize the reordering strategy and proposed a semantic analysis method to avoid introducing new bugs.
- We evaluate ESfix in the context of detector-reported bugs, and ESfix automatically repairs most real-world concurrency defect bugs (92.6%) with performance and simplicity similar to manual verification.

## 2. Background and Motivation

### 2.1. Example Program

**Figure 1:**
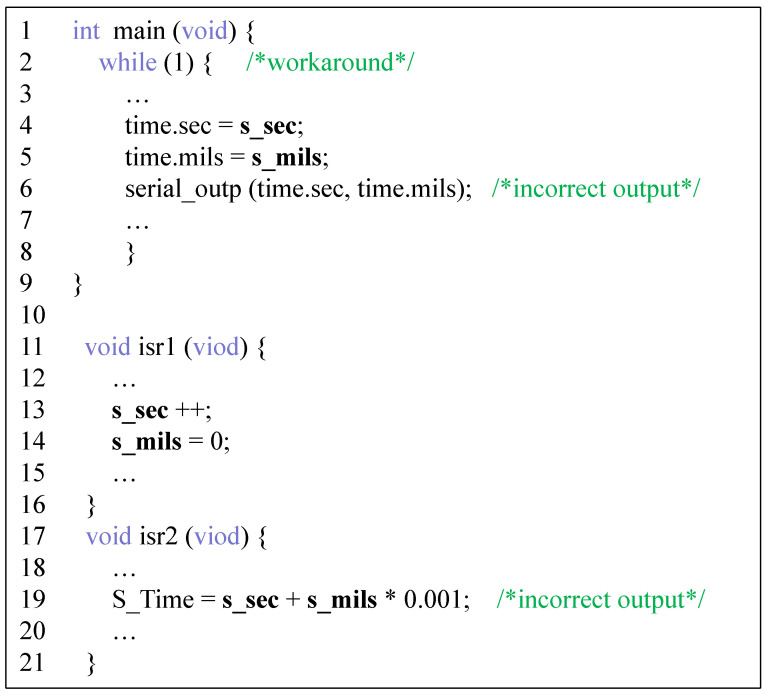
Example program.

Figure 1 shows a simplified example program fragment. It is a typical interrupt driver program consisting of a main program and several interrupt service programs, with s_sec and s_mils denoting shared variables. The main() function obtains the seconds and milliseconds data for the current global clock. The function isr1() is a timer ISR that updates the s_sec and s_mils information at a fixed time. The function isr2() calculates the current time by reading these two values, where isr1() has a higher priority than isr2(). Since ISRs are not disabled in the task, if an interrupt is triggered during the execution of the task, the execution pointer of the CPU will jump to the entry of the corresponding ISR, interruptive concurrency occurs, and the shared variables are preempted and modified. For example, isr1() may preempt and modify the shared variables s_sec and s_mils (lines 4–5), causing the port to receive erroneous information (line 6). To prevent this race condition from occurring, the computation process in the main() function needs to be uninterruptible, meaning that interrupts should be disabled at this point. That is, repairing an interrupt-related race condition requires enforcing interrupt-specific synchronization operations. For example, between lines 4 and 5, the interrupt disable operation associated with isr1() should be inserted before access to line 4, and the interrupt associated with isr1() should be enabled after access.

### 2.2. Challenges

**Figure 2:**
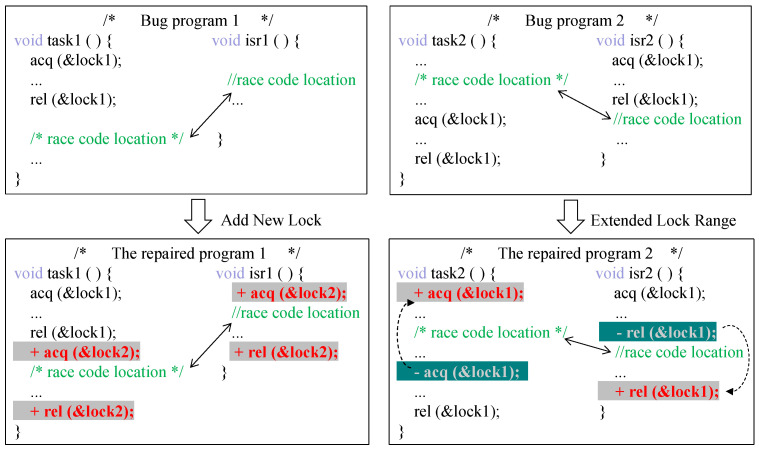
Example of adding a lock policy.

In previous research on concurrency defect repair, most research has automatically created or extended critical regions protecting shared variables by adding locking strategies, conditional variables, etc., and repairing them by adding new locks or extending lock ranges as shown in Figure 2 (the choice between these two types of repair depends on which resources in the program are protected by locks, that is, which rows constitute the critical region). However, repair techniques may produce large critical regions protected by lock operations, which may introduce performance bugs in the repaired program. For example, adding globally protected locks to a program’s existing lock protection may result in deadlocks.

**Figure 3:**
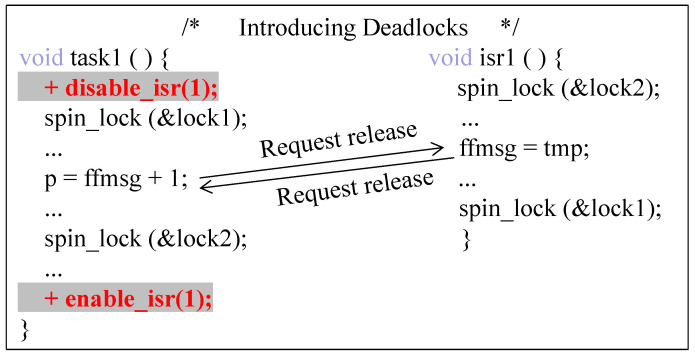
Adding disabled interrupts causes deadlocks.

Another method is to use the disable/enable interrupt operation to force the execution order of the two threads (task and ISR), but this may introduce new concurrency defects. As shown in Figure 3, when a task holds a mutually exclusive shared resource (such as spin locks, semaphores) while disabling interrupts, the interrupt handler needs to hold the resource as well. At this point, the interrupt must always wait for the task to complete its access and release the resource, but since the task holding the resource must also wait for the interrupt to return, this leads to a deadlock.

When atomicity violation defects are repaired, reordering strategies or lock protection strategies are generally used. Reordering is generally achieved by moving the write statement forward, realizing the execution of the operation within the same thread before executing the operation in another thread. However, when the write statement is moved, the value of the stored variable may change prematurely, which leads to the change of the value after the moved statement. Also, when repairing concurrency defects, it is necessary to consider how to make the repair as reasonably simple as possible and minimize excessive overhead while ensuring that the repair is correct.

## 3. Method

In this section, we introduce a concurrent defect repair method called ESfix. We introduce the overall framework of the method. We detail how to find the insertion location of an interruption strategy, and how to select a locking strategy and adjust the repair range. Also, we use the reordering strategy to solve the semantic inequality problem in atomicity violation repair. Thereby, the repair accuracy and efficiency are improved.

### 3.1. Overall Framework

**Figure 4:**
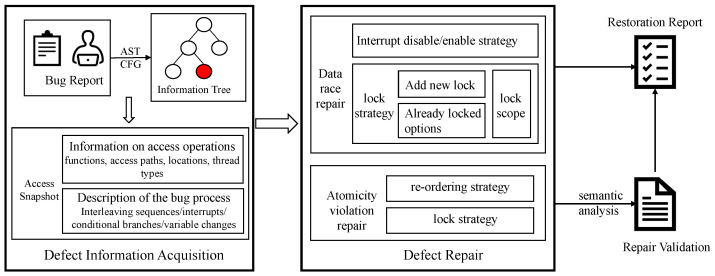
ESfix working framework.

The complete structure of ESfix is shown in Figure 4, which consists of three steps. (1) Defect information acquisition. We use mutual information to analyze the dependencies between codes, locate the source line code and context where concurrent defects occur through static analysis in the Abstract Syntax Tree (AST), and generate a ranked list of suspicious code locations that must be modified based on event information. (2) Generate repair strategies. For each validated concurrency defect warning, we guide the planning of specific repair methods through “synchronization” strategies, reordering strategies, and interrupt disable/enable strategies. Since this stage may introduce unnecessary synchronization, we use an optimization algorithm to remove any redundant synchronization to reduce the repair operation to lower the performance overhead. (3) Repair Validation. Since the repair location is close to the suspicious code point location, the repaired program needs to be validated to ensure that the repair meets the expected behavior of the code in the embedded program. We use IntRace [23], TSAFL [24], and manual code review by developers to validate the repaired programs, and quantify and evaluate the effectiveness of bug repairs through information entropy.

### 3.2. Interrupt Disable/Enable Strategy

The interrupt disable/enable strategy automatically enforces insertion operations on tasks or ISRs to avoid triggering interrupts that may lead to concurrency defects. ESfix decides where to insert interrupt disable and interrupt enable operations by analyzing where shared resources are acquired and released. Given a race pair $\{<e_{p}=\left(T_{i},W_{i},Op_{i}\right)>$,$<e_{c}=\left(T_{j},W_{j},Op_{j}\right)>\}$, if $e_{p}$ inserts a disable interrupt operation before $e_{c}$ waits for the mutually exclusive shared resource to be released, a deadlock may be triggered. Therefore, the repair strategy should ensure that there is no mutually exclusive shared resource between $I_{p}$ and $T_{j}$.

Equation (1) defines a sufficient condition for inserting an interrupt disable operation before operating instruction $I_{p}$ while avoiding deadlock:

(1) $$Mutua\left(I_{p}\right)\cap \left(\underset{C\in W_{T_{j}}}{⋃}Mutua\left(c\right)\right)=Ø$$

where $I_{p}$ is an access operation instruction of $T_{i}$ at the location where the interrupt disable operation is inserted, and $W_{T_{j}}$ denotes the set of all operations in $T_{j}$. Mutua(x) denotes all possible mutually exclusive shared resources that can be accessed and released by the operation *x*.

Given an operation instruction location *x*, ESfix performs the following operations: (1) We analyze all mutually exclusive shared resources held by instruction *x* and add them to the set. (2) We add mutually exclusive shared resources to the Mutua(x) when and only when they are acquired before *x* and released after *x* according to the control flow graph information from the static analysis state. (3) We compare the time regions and determine the condition when the regions where the mutually exclusive shared resources are acquired or released in $T_{i}$ and $T_{j}$ are non-overlapping.

Subsequently, we determine the instruction locations $L_{f}$ and $L_{p}$ at which the interrupt disable and enable operations are inserted. Let $L_{i}$ be the instruction operation located at location $W_{i}$, and ESfix searches for the appropriate instruction location along the control flow graph forward of $L_{i}$, starting at location $L_{i}$. Given a location $L_{f}$, ESfix performs the following checks: (1) If the instruction $L_{f}$ belongs to all of $L_{i}$’s predecessor instructions (including $L_{i}$), the search continues. (2) If the minimum number of instructions from the instruction $L_{f}$ to $L_{i}$ is not greater than the distance from any of the other predecessor instructions to $L_{i}$, it verifies whether deadlock will be generated. (3) It is necessary to ensure that $L_{f}$ satisfies Equation (1). lf $L_{f}$ satisfies these conditions, it is selected as the point at which to insert the interrupt disable:

(2) $$\forall L_{f},L_{x}\in Front\left(L_{i}\right)\wedge Min\_inst\left(L_{f},L_{i}\right)\leq Min\_inst\left(L_{x},L_{i}\right)$$

After finding $L_{f}$, we analyze $T_{i}$ in order to find the instruction $L_{p}$, which is conditioned by Equations (3) and (4):

(3) $$\forall L_{p}\in Post\_contr\left(L_{f}\right)\cap L_{f}\in Contr\left(L_{p}\right)$$

(4) $$\forall L_{y}\in Post\_contr\left(L_{f}\right)\wedge Min\_inst\left(L_{f},L_{p}\right)\leq Min\_inst\left(L_{f},L_{y}\right)$$

In other words, ESfix starts from the $L_{f}$ previously identified and searches backward along the CFG instruction of $L_{f}$ to the appropriate location of $L_{p}$. Post_contr() denotes that $L_{p}$ dominates $L_{f}$ after $L_{p}$, and contr() denotes that $L_{f}$ dominates $L_{p}$, Min_inst() denotes the minimum distance, and $L_{p}$ is the closest position after $L_{f}$. In this way, we can easily ensure that an interrupt disable operation is inserted before $L_{f}$ and an interrupt enable operation is inserted after $L_{p}$.

**Patch generation example.** Example program as in Figure 1, for data race in lines 4–5:

- Static analysis: identify the access points of the shared variables s_sec and s_mils and confirm that they are located in a non-atomic operation region.
- Interrupt operation insertion:
  Insert disable_isr(1) before access (disable isr1 interrupt before line 4).
  Insert enable_isr(1) after access (enable isr1 interrupt after line 5).
- Deadlock avoidance: verifying that there is no overlap of mutually exclusive resources between insertion locations $L_{f}$ and $L_{p}$ through Equation (1) to ensure that no task blocking is caused by interrupt disabling.

### 3.3. Lock Selection Strategy

In general, concurrency defects can occur due to programmers forgetting lock protection or using locks incorrectly. In addition to interrupting the disable/enable repair strategy, concurrency defects are usually avoided by using generating new locks and protecting affected memory accesses by the same lock object, care needs to be taken when adjusting lock scopes that deadlocks should not be introduced. Second, it is equally important to deal with protection ranges, where multiple locks can be merged into a single lock to produce a succinct repair.

Algorithm 1 shows the implementation of the lock strategy selection. Data race involves at least two accesses to a set of shared variables that may be protected by some locks but may also have no locks protecting the associated accesses. Therefore, it is not always necessary to introduce new locks to serialize threads to repair concurrency defects. We analyze a given concurrency defect according to this idea to identify all lock acquisitions involved and then explore the use of locks.

For the case where there is already a lock in the program, we define the lock priority rule (Equation (5)) to select the optimal lock by calculating the lock priority.

**Rule 1.** If the return value is 1, the lock $S_{1}$ has high priority, and Algorithm 1 prioritizes this lock for repair:

(5) $$prior\left(S_{1},S_{2}\right)=\left\{\begin{array}{c} 1,N_{lock}\left(S_{1}\right)>N_{lock}\left(S_{2}\right)\\ 1,global\_lock\left(S_{1}\right)>class\_lock\left(S_{2}\right)\\ 1,CoverO\left(S_{1}\right)>CoverO\left(S_{2}\right)\\ 0,other \end{array}\right.$$

**Algorithm 1** Lock strategy selection algorithm**Input:** Data race pairwise sets *C*,AST
**Output:** Repair strategy $\hat{f}$
  1:$\hat{f}\leftarrow Ø$;  2://Check if the variable related to the defect has been locked-protected  3:**for** $<e_{p},e_{c}>$ in *C* **do**  4:      **if** Shared_var←Lock() **then**  5:            $f\leftarrow choice(prior\left(S_{1},s_{2}\right))$; //Choose which existing lock to use according to Rule 1  6:            Modify_lock_range();  7:      **else**Shared_var←unLock()  8:            Find_AST_Info();  9:            lock←CreateNewLock();10:            var←freshvariablename;11:            act←DECLARE(cls(B),var);12:            $\hat{f}\leftarrow var$;13:     **end if**14:**end for**15:**return** $\hat{f}$


$S_{1}$,$S_{2}$ are two locks in the program, $N_{lock}\left(S_{i}\right)$ denotes that $S_{i}$ is the lock that protects the most shared variables, thus avoiding nested synchronization, and $CoverO(S_{i})$ denotes the range of operations covered by the lock $S_{i}$. Meanwhile, if the variables involved are all global, a global lock is sufficient. If the variables involved are fields of the same class or structure, a field lock in the same class/structure is sufficient.

After we confirm the chosen lock, the lock range is adjusted according to the following two scenarios, with the criterion that there must be the same lock to protect at least one access from each thread:

- For a data race pair $e_{p},e_{c}$, the lock for the rest of the thread where $e_{p}$ is located is the same as the lock protecting $e_{c}$ in the other thread. We can extend the lock scope of the thread, in which $e_{p}$ is located to include the $e_{p}$ operation by extending its scope.
- For a data race pair $e_{p},e_{c}$, there is an overlap of the critical regions protected by locks in $e_{p}$ and $e_{c}$, and there are no other instructions in the critical regions. We can merge the critical regions and replace both locks using the same lock to reduce the number of repair operations.

In addition, if there is no lock that can protect any access operation from data race, we introduce a new lock. When introducing a new lock, the order of the locks introduced (if any) must be handled carefully to avoid introducing deadlocks. The new lock is represented using the following simple domain-specific types, using global variable locks Insert_Lock(lock), field locks Field_Lock(class,var), and synchronization locks SYNC(A,lock), respectively, depending on the context node associated with the shared variable:

(6) $$\hat{f}::=Insert\_Lock\left(lock\right)\left|Field\_Lock\left(class,var\right)\right|SYNC\left(A,lock\right)$$

**Rule 2.** We record the functions and instructions related to the race operation and locate the source code through the Abstract Syntax Tree (AST). ESfix examines the first node and the last node associated with the variable and determines the critical region $\left[Critical\left(S_{x}\right),Critical\left(S_{y}\right)\right]$ where the race occurs:

(7) $$\forall Critical\left(S_{x}\right),Critical\left(S_{y}\right)\in Critical\left(S_{i}\right)$$

(8) $$Critical\left(S_{x}\right)⊏First\_Node\left(var\right)|Critical\left(S_{y}\right)⊏Last\_Node\left(var\right)$$

(9) $$REPLACE(Critical\left(S_{x}\right),Critical\left(S_{y}\right))$$

Algorithm  2 aims to infer new locks from the set of bugs, and for each set of bugs, the algorithm analyzes the AST operation (line 2) to derive a public lock parameterized by a tree node that ensures that all its accesses are protected (lines 3–9). To solve the self-deadlocking problem, which is caused by the repeated acquisition of the same lock by the same thread, the algorithm changes the properties of the newly introduced locks to re-entrant locks (line 11) since re-entrant locks allow the same thread to acquire and release locks multiple times in a nested fashion. To avoid introducing new lock sequences, the algorithm chooses to keep the original lock acquisition strategy. This is because the original lock acquisitions may be scattered in different parts of the program, especially in functions that can be called from multiple branches of control. Removing these locks may break the original synchronization logic of the program and lead to unforeseen concurrency problems.
**Algorithm 2** Inferring new locks from bug sets**Input:** Bug Set *P*,AST
**Output:** newly added lock $\hat{f}$
  1://Minimum scope of operations related to shared variables for locating the bug set  2:$No.i\leftarrow <e_{p},e_{c}>$;  3:**switch** ($\hat{f}$)  4:**case** variables are global**:**  5:         $Insert\_Lock(\hat{f})$;  6:**case** variables are class or structure fields**:**  7:         $Field\_Lock(\hat{f})$;  8:**case** use synchronization**:**  9:         $Sync(\hat{f})$;10:         $Replace(\hat{f})$;11:         Lock before the critical area associated with the variable $Critical(S_{i})$, release the lock after $Critical(S_{i})$;12:**return** $\hat{f}$


### 3.4. Reordering Strategy

Atomicity violations involve at least three accesses to a set of shared variables, and are usually repaired by inserting new locks to serialize all executions of the threads involved in the concurrency bug. The principle of repair is to move two access operations of the same thread to be executed before the access operation with which the atomicity violation bug occurs in another thread.

**Figure 5:**
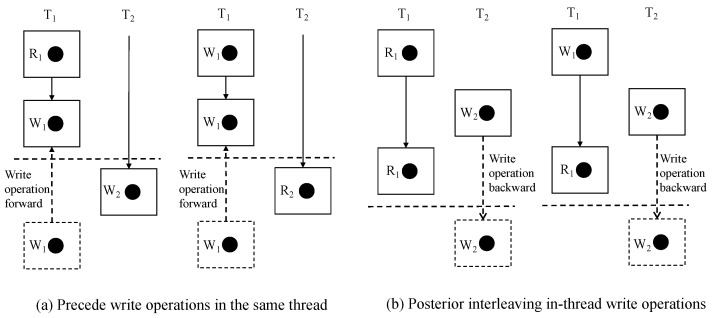
Two repair strategies.

The specific method is shown in Figure 5. There are two cases where the four atomicity violation types need to be sorted in the interleaved access space. For the type of forward same thread write operation, it is necessary to move the write access operation within the same thread ($T_{1}$) to before the read/write operation in another thread ($T_{2}$) so that it waits for the two access operations in the same thread to be executed first. And for the type of write operation in the post-interleaved thread (another thread), it is necessary to delay the write operation in the interleaved thread so that it waits for the two access operations in the same thread to be executed first, and then $T_{2}$ starts to be executed when the atomic access region is executed.

In general, the same thread’s atomic access region and the intertwined thread’s shared variable access operation will jointly obtain the required information (such as bank deposit/withdrawal and global time acquisition), and if we blindly reset the order of the access operation, it may result in the problem of different semantics. Therefore, we use Clang to build CFG descriptions to extract read/write dependencies and conditional expressions in individual paths. ESfix analyzes the connectivity of the repaired paths by determining three conditions, whether the inputs have changed, whether the connectivity has changed, and whether a new valid path has been generated, thus solving the semantic problem in the reordering strategy.

## 4. Experiments

To evaluate correctness, we manually check each repair to determine if it repairs the bug and if it changes the original program semantics. To evaluate performance, for each detected bug, we collect the execution time and runtime overhead (i.e., performance impact) of the repair. We use the average execution time to compute the overhead.

The experiments aim to answer the following research questions:

- RQ1: How does ESfix compare to other repair tools in terms of effectiveness and performance of atomicity violation repair?
- RQ2: How does ESfix perform in repairing the data race on industrial embedded projects?

### 4.1. Experimental Setup

Datasets. For repairing atomicity violations, we use concurrent bugs in embedded software and concurrent bugs in general-purpose software as datasets, respectively, as shown in Table 1.

The embedded software test set contains 6 atomicity violation program packages [25,26]. Each package contains 3 programs, for a total of 18 real embedded programs. Specifically, “logger” is the program that models the firmware part of a temperature logging device in a major industrial enterprise. The “blink” controls the LEDs connected to the MSP430 hardware, checks the timer value, and changes the LED blinking according to the timer value. The “brake” is the program generated from the MATLAB/Simulink model of the Volvo Technology’s line control actuator system. The “i2c_pca_isa”, “i8xx_tco” and “wdt_pci” are from the Linux kernel driver for the hardware support of the ISA board, TCO timer, and watchdog for the i8xx chipset.

The generic software dataset contains two sets of real concurrency bugs. The first set is a bug-repair benchmark suite built by CFix [27], which contains 13 concurrency bugs, most of which contain tens to hundreds of thousands lines of code that can lead to serious crashes and security vulnerabilities. These bugs come from public releases of 10 open-source C/C++ multi-threaded applications, and are representative of benchmarks used in many previous works. We select six benchmarks that include atomicity violation bugs. The second set [28] is a real-world benchmark set consisting of 20 benchmarks, of which we exclude 11 of them: 1 deadlock, 1 re-entrant bug, 3 order violations, 1 atomicity violation involving Java code, 3 duplicates of the first set, and 2 not compiling correctly, for a total of 9 benchmarks.

For the repair of the data race, we choose real cases on industrial embedded software as the benchmark for evaluation. As shown in Table 2, lines 1–4 are device drivers in the Linux kernel and LDD. Lines 4–10 are representative test cases from the independent test bug database of the China Academy of Space Technology (CAST), and the size of the selected software ranges from 99 to 5099 lines. This is typical of industrial interrupt drivers.

Regarding comparison methods, many approaches have been proposed to repair concurrency bugs, but only a few target C/C++ programs. Therefore, when evaluating atomicity violation repair, we choose Baseline and αFixer [29] as comparison methods. Baseline is the baseline of ESfix, which uses traditional generation and verification methods without using static type analysis and semantic equivalence detection. αFixer determines the visibility of variables, inserts different levels of gate locks or adjusts lock ranges to repair atomicity violations.

After applying these comparison methods to all benchmarks, we run each fixed program 10 times with each method and collect the results. In these 10 runs, we insert a set of random sleeps before and after each lock acquisition of the fixed program to amplify the probability of any introduced deadlocks occurring. The execution time of the repair without sleeps is also counted. To evaluate the performance scalability, the experiments set the number of threads to 2, 4, 8, 16, 32, 64, and 128, amplifying the overhead introduced by each technique for comparison.

When evaluating the atomicity violation defect dataset and the data race dataset containing interrupted programs, which should be used on embedded software, we arrange three experienced programmers to independently perform manual verification. First, a code review is performed to ensure that the repair logic matches the root cause of the defect (e.g., data race or atomicity violation). Second, the correctness of the repair procedure is verified by the static analysis tools IntRace [23] and TSAFL [24]. If the tools fail to effectively analyze the correctness of the repair, the three programmers will perform manual repair and compare the repair results on a real industrial embedded project and calculate the repair rate. All repairs are cross validated to ensure consistency (if there is disagreement between the three results, a fourth programmer arbitrates).

### 4.2. Generalized Dataset Atomicity Violation Repair Analysis

This section compares the experimental results of the proposed method with Baseline and αFixer in Table 1 with the generalized dataset. Table 3 summarizes the repair results of the three methods for all 15 benchmark tests. Overall, Baseline has a repair rate of 35.6% (2 complete and 9 partial repairs), αFixer has a repair rate of 69.2% (5 complete and 9 partial repairs), and ESfix has a repair rate of 92.6% (13 complete and 1 partial repair).

In addition, in the performance scalability test with 128 threads, Baseline has the largest average overhead of 85.9%, followed by αFixer with an average overhead of 29.4%. ESfix has an average overhead of only 11.8%. These results show that ESfix outperforms the other techniques in terms of effectiveness and efficiency.

#### 4.2.1. Repair Effectiveness

Table 4 shows the detailed repair results of the three methods on the dataset. Columns 1–2 show the benchmark information, and column 3 shows the total number of atomicity violation bugs in the benchmarks. The remaining columns show the number of correctly repaired bugs (#C), deadlocks introduced (#D), and repair rate for each method. In the last row, we show the total number of repairs, the deadlocks introduced, and the average repair rate.

In the effectiveness evaluation, the number of successfully repaired bugs is a common evaluation metric. We view the process of bug repairing as a process of reducing system uncertainty, that is, lowering the entropy of the system. The repair rate can be defined as the ratio of the number of correctly repaired errors to the total number of detected errors, which actually reflects the system’s transition from a higher entropy state (with more unknown bugs) to a lower entropy state (bugs identified and repaired). The higher the ratio, the more the system uncertainty is reduced, that is, the more bugs repaired:

(10) $$F_{Rate}=\frac{\#C}{\#B}=\frac{n(CorrectRepair)}{n(CorrectRepair+WrongRepair+InvalidRepair)}$$

It can be observed from Table 4 that Baseline performs incomplete repair in 11 programs, of which the repair rate is less than 50% in 7 programs, which indirectly proves the necessity of static type analysis and semantic equivalence detection. αFixer can infer lock visibility but cannot check semantic equivalence, and although some repair is performed in all 14 programs, atomicity violation bugs are completely repaired in only 5 programs, with the repair rate of the remaining programs ranging from 40% to 83%. ESfix makes complete repairs on 13 programs, and the repair rate on another program reaches 89%. None of the three methods complete the repair of Mozilla-142651 on 15 programs, which we manually examine and find that the atomicity violation in the program is benign and therefore cannot be repaired.

During the repair process, Baseline and αFixer introduce 7 and 3 deadlocks, respectively, while ESfix does not introduce any deadlocks. The locks introduced by Baseline are all self-deadlocks, and in MySQL-12848, Baseline protects qSize write operations by expanding the lock range of the gMutex. However, this lock range is too large, and the thread also contains a pair of lock acquisitions/releases, which can lead to blocking on lock acquisitions and thus deadlocks. On the Mozilla-18025 program, while the αFixer changes the lock’s attribute to re-entrant and correctly extends the lock’s range, it does not consider whether there are changes between the conditional branching statement and the dependent storage, which creates new path information and causes a deadlock.

ESfix can correctly repair this atomicity violation. First, ESfix tries to repair it by front-loading the write operation, storing the shared variable value into a local variable, and checking that the modified statement equivalence has not changed. Then, ESfix discovers that the function call in the second write operation contains this lock release/acquisition pair, and it avoids this potential self-deadlock by changing the lock’s properties.

#### 4.2.2. Repair Efficiency Comparison

**Figure 6:**
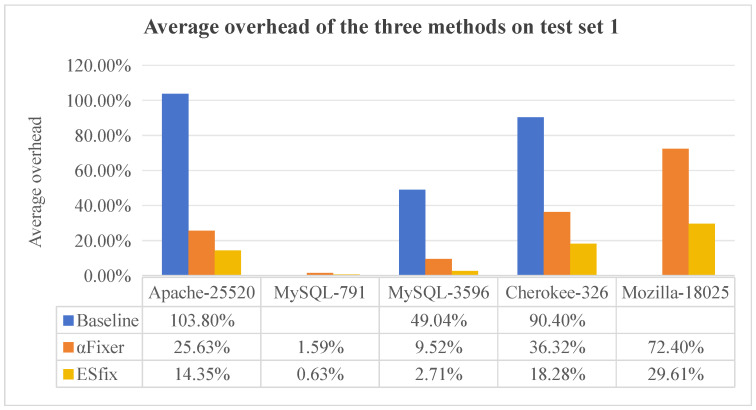
Comparison of repair efficiency of three methods on test set 1.

**Figure 7:**
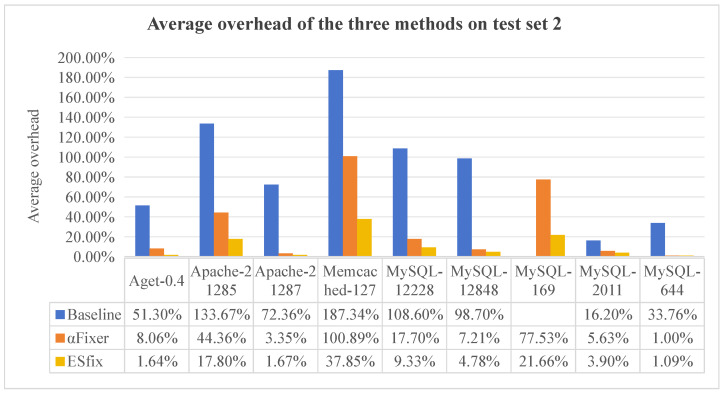
Comparison of repair efficiency of three methods on test set 2.

Figure 6 and Figure 7 show the average overhead for each method for a thread count of 128, with blanks indicating that the method does not repair the program (0.0% repair rate) and therefore no data are collected. Overall, ESfix generates significantly lower overhead than Baseline and αFixer, with Baseline generating the largest overhead, especially in Memcached-127, where it reaches 187.34%. In benchmarks where all three methods are handled correctly, αFixer and ESfix introduce almost the same overhead, while Baseline is much higher than both methods. This is because Baseline always introduces global locks, whereas αFixer and ESfix can infer lock visibility and scope-specific lock types, respectively, which does not introduce additional overhead.

### 4.3. Embedded Software on Atomicity Violation Repair Analysis

To comprehensively evaluate the effectiveness of ESfix in repairing atomicity violation problems in embedded software, this section applies ESfix to each atomicity violation program package. To ensure the accuracy and reliability of the results, ESfix is applied to each program 10 times, and the average integer repair is taken as the final measurement.

Table 5 shows the repair results of ESfix, with columns 1–3 showing the benchmark information, where #LOC denotes the code function and #SV denotes the number of shared variables contained in the program. Column 4 indicating the total number of atomicity violation bugs for each benchmark. The other columns show the number of correctly repaired bugs (#C), repair failures (#F), and deadlocks introduced (#D), and finally the repair rate is calculated on each test case. We manually check the correctness of each actual atomicity violation repair in each program, and the bug is considered repaired if the atomicity violation is successfully eliminated without introducing a new bug.

The experimental results show that ESfix achieves complete repair on two packages, brake and i8xx_tco, and the repair rate on the rest of the packages reaches more than 85%. Overall, the average repair rate is 93.7%, and ESfix can effectively identify and repair atomicity violations in most cases. At the same time, no deadlocks are introduced during the repair process. An in-depth analysis of the three bugs in the wdt_pci package that are not correctly repaired shows that the cause of the failures is the incorrect location of the stored variables, which results in moving the wrong statements.

### 4.4. Data Race Repair Analysis

We systematically apply ESfix to each program containing data race and manually check the correctness of the data race repair in each program, and consider the bug repaired if it is successfully eliminated without introducing a new bug. ESfix is applied to each program 10 times, and the average time is taken as the final measurement.

Table 6 shows the repair results of ESfix, with columns 1–2 showing the benchmark information, column 3 showing the number of data races (#Race) that the program contains, and column 4 showing the repair strategy. In this table, $Int_{x}+Lock_{y}$ indicates that ESfix repairs the benchmark using x interrupt enable/disable strategies or y lock strategies. The other columns of the table show the number of deadlocks introduced by the repair (#D) and whether the repair is successful.

The results of the research show that the repair of all 10 programs is effective and does not introduce deadlocks. ESfix correctly repairs all detected bugs and gives the preferred repair method. For example, for module5 of the program, it contains 3 interrupts, 30 shared variables, and a total of 13 data race bugs. We use two methods to repair it, either by adding 3 pairs of interrupt strategies (disable_isr( ) and enable_isr( )), or using lock strategies, which although containing 30 shared variables, not all of them have data race bugs. The locking strategy collects information about access summaries and orders the locks by priority, and ESfix eventually introduces a new global locking variable and inserts 20 pairs of locking/unlocking operations before and after the relevant statements. The number of repair operations is not very high, even for programs with many race. The main reason for this is that we optimize the lock range and select the optimal locks through the preference strategy.

**Figure 8:**
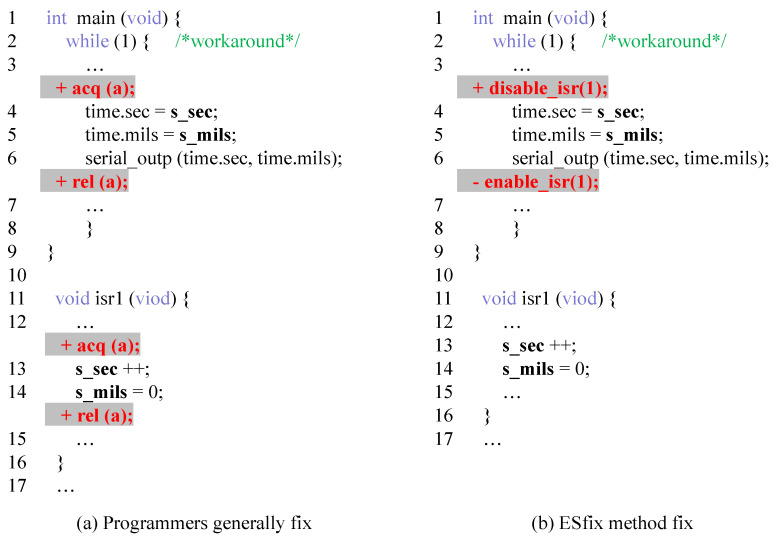
Simple example of data race defect fixing.

Figure 8 shows a simple example of a data race defect repair in the program of Figure 1, showing only the key parts. A high-quality repair should choose fewer lock-acquiring/releasing or interrupt-disabling/enabling operations, and the range of locks should be as small as possible. This produces a program that does not result in a race on the shared variables (in addition, it needs to be ensured that it does not create deadlocks with the already existing lock acquisitions/releases) and excessive overhead. Figure 8a shows a general program repair strategy and Figure 8b shows our strategy. In contrast, our repair strategy can lock more precise ranges while using synchronization formats more similar to what the programmer might have written.

## 5. Related Work

Automated Program Repair (APR) methods have been constructed to help developers reduce the time and effort spent repairing software defects. By deploying them at the source code level, they locate where repairs can be applied and make the necessary adjustments to repair the defects, thus preventing further bugs caused by the same defect. Currently, most concurrent defect repair methods rely on semantics-driven methods and generation-verification methods (G&V).

### 5.1. Semantics-Driven Methods

Semantics-driven methods [11,12,13] implicitly or explicitly encode problem preprocessing, and once the correct program specification is found, it is used as a constraint to guide the process of program repair or to validate the correctness of the repair. S3 [12] collects the constraint information from symbolic execution as constraint solver’s inputs, which ranks the patches by comparing the syntactic and semantic similarities of the code before and after the modification, and considers the syntactic and semantic features of the program to guide the patch generation process. FAngelix [13] performs a random search for constraints using Markov Chain Monte Carlo (MCMC) sampling and selects the repair that is syntactically or semantically closest to the original program being repaired by converting it to the execution path closest to the one observed in the original program but is not able to deal with programs that contain infinite loops. Also, semantics-driven methods usually deal with specific types of bugs rather than generic ones.

### 5.2. Generation-Verification Methods

The generation-verification method [30,31,32] uses a series of mutation operations to modify the defective program and evaluates the correctness of the patch using test cases as criteria and consists of three basic processes: fault location (FL) [33,34] to determine the location of potential programs that may cause bugs; patch generation (PG) generates a set of candidate patches that implement change operators applied to code locations; and patch verification (PV) [35] executes test cases to ensure that the patches meet the expected behavior of the code in the test suite. In the patch generation phase, search-based, template-based, and learning-based patch generation techniques are typically used.

#### 5.2.1. Search-Based

Search-based patch generation techniques [36,37,38] apply change operators randomly or are guided by heuristic or metaheuristic search algorithms. For example, ARC [37] uses a crossover-free genetic algorithm to mutate incorrect programs, searching for variants of the original program that repair deadlocks and data race. SCRepair [38] uses random mutation to generate candidate patches and selects the patch that passes all test cases as the correct one. However, they suffer from path space explosion.

#### 5.2.2. Template-Based

Template-based patch generation techniques [39,40,41,42,43] rely on some predefined repair templates based on the developer’s or researcher’s experience, and then retrieve reusable code snippets matching the repair templates from the local codebase to repair the defective program. HFix [41] introduces a move operation by rearranging the location of memory access statements and synchronization operations that are already present in the same thread, instead of adding a new synchronization to repair atomicity problems. sGuard [42] and Elysium [43] have designed templates for adding locks at the source and bytecode levels, respectively. However, the lock templates used in sGuard modify the lock storage variables for each re-entrant function, resulting in higher overhead costs. These methods offer higher controllability but are limited by template diversity and editorial expressiveness, and in practice require significant effort and expertise to produce.

#### 5.2.3. Learning-Based

Learning-based patch generation techniques [9,44,45,46,47,48] typically view program repair as a Neural Machine Translation (NMT) task, training deep learning models to capture bug context and generate patches for defective programs, converting defective programs to stationary programs. CURE [45] pre-trains an NMT model on a large corpus of developer code and uses a static checking strategy to generate patches with valid identifiers which improves syntactic correctness. AlphaRepair [47] uses mask tokens to replace defective code to anchor the repair range of the pre-trained language model, and then generates patches using the Masked Language Model (MLM). Confix [48] extracts the necessary information based on the semantic context of the AST nodes, and assists the pre-trained model in generating the correct patch by introducing a repair strategy that accomplishes the defective program’s repair. However, the above methods face the impact of training data quality and diversity, leading to a large number of bugs and redundant code generation.

## 6. Conclusions

We propose a repair method for concurrent defects in embedded software called ESfix, aimed at improving software security and stability while reducing the time and effort required to repair software defects. ESfix is specifically designed for embedded software environments, which can accurately locate the code areas that need to be repaired and extract node information of relevant defect codes. This method solves the data competition problem by optimizing the interrupt disable/enable strategy and lock mechanism, and adopts a reordering repair strategy to repair atomic violation defects, ensuring the integrity and consistency of information. Through experimental evaluation, ESfix has demonstrated the potential to outperform other technologies in terms of repair effectiveness and efficiency, effectively reducing entropy in software systems, improving information predictability and reliability, and thereby reducing uncertainty and potential errors in system operation.

## Figures and Tables

**Table 1:** Information about the atomicity violation dataset.

No.	Name	Describe
1	logger	Procedure for modeling a portion of the firmware of a temperature recording device in a large industrial enterprise.
2	blink	Program to control LEDs connected to MSP430 hardware.
3	brake	Used to calculate brake torque based on the speed of each wheel.
4	i2c_pca_isa	Programs collected from Linux kernel drivers to support hardware ISA boards.
5	i8xx_tco	Programs collected from Linux kernel drivers to support TCO timers for the i8xx chipset.
6	wdt_pci	Programs collected from Linux kernel drivers for watchdogs.
7	Generalized Dataset 1	CFix-built bug-repair benchmark suite with 6 atomicity violation benchmarks from open-source C/C++ multi-threaded applications.
8	Generalized Dataset 2	The real-world benchmarks used in the previous methodology had a total of nine programs that contained atomicity violation bugs.

**Table 2:** Information related to data race datasets for real programs.

No.	Name	Loc.	Describe
1	mv643xx_eth	3256	A device driver in the Linux kernel.
2	short	704	A driver in LDD.
3	shortprint	531	A driver in LDD.
4	mpu401_uart	630	A device driver in the Linux kernel.
5	module1	168	A Lower Disk Driver.
6	module2	154	An engine power control software.
7	module3	99	A thermal control software.
8	module4	1710	A power control software.
9	module5	2273	CA battery supply control software.

**Table 3:** Overall restoration results for the three methods.

Program	Atomicity Violation Repair Rate			Average Expenditure		
	Baseline	αfixer	ESfix	Baseline	αfixer	ESfix
15	35.6%(11)	69.2%(14)	92.6%(14)	85.9%	29.4%	11.8%

**Table 4:** Comparison of the restoration results of the three methods on the dataset.

Name.	Size(Loc)	#B	Baseline				αfixer				ESfix		
			#C	#D	Fix Date		#C	#D	Fix Date		#C	#D	Fix Date
Apache-25520	333K	6	2	1	33.3%		5	0	83.3%		6	0	100.0%
MySQL-791	125	2	0	0	0.0%		1	0	50.0%		2	0	100.0%
MySQL-3596	122	4	1	0	25.0%		4	0	100.0%		4	0	100.0%
Mozilla-142651	87K	1	0	1	0.0%		0	1	0.0%		0	0	0.0%
Cherokee-326	83K	5	3	1	60.0%		4	1	80.0%		5	0	100.0%
Mozilla-18025	108K	2	0	1	0.0%		2	1	100.0%		2	0	100.0%
Aget-0.4	320	2	1	0	50.0%		1	0	50.0%		2	0	100.0%
Apache-21285	45.34K	9	4	1	44.4%		7	0	77.8%		8	0	88.9%
Apache-21287	45.61K	7	3	0	42.9%		4	0	57.1%		7	0	100.0%
Memcached-127	1.27K	5	1	0	20.0%		2	0	40.0%		5	0	100.0%
MySQL-12228	122	4	1	1	25.0%		2	0	50.0%		4	0	100.0%
MySQL-12848	181	2	2	1	100.0%		2	0	100.0%		2	0	100.0%
MySQL-169	145	4	0	0	0.0%		4	0	100.0%		4	0	100.0%
MySQL-2011	126	6	2	0	33.3%		3	0	50.0%		6	0	100.0%
MySQL-644	118	1	1	0	100.0%		1	0	100.0%		2	0	100.0%
SUM		60	21	7	35.6%		42	3	69.2%		58	0	92.6%

**Table 5:** ESfix results of the atomic violation dataset in the embedded software.

Name	#LOC	#SV	#Race	#C	#F	#D	Repair Rate
logger	578	46	21	19	2	0	90.5%
blink	373	57	26	25	2	1	96.2%
brake	1975	130	7	7	2	0	100.0%
i2c_pca_isa	1063	20	20	18	2	0	90.0%
i8xx_tco	2671	60	2	2	0	0	100.0%
wdt_pci	3513	61	21	18	3	0	85.75%

**Table 6:** ESfix results on data race.

No.	Name	#Race	Strategy	#D	Repair Status	Time (s)
1	mv643xx_eth	10	$Int_{20}+Lock_{22}$	0	success	54.62
2	short	18	$Int_{14}+Lock_{26}$	0	success	16.07
3	shortprint	0	N/A	N/A	success	N/A
4	mpu401_uart	47	$Int_{28}+Lock_{44}$	0	success	108.75
5	module1	4	$Int_{2}+Lock_{4}$	0	success	5.93
6	module2	64	$Int_{10}+Lock_{16}$	0	success	13.43
7	module3	12	$Int_{4}+Lock_{12}$	0	success	9.87
8	module4	9	$Int_{4}+Lock_{28}$	0	success	21.40
9	module5	13	$Int_{6}+Lock_{40}$	0	success	58.92

## Data Availability

Data are available in a publicly accessible repository.
